# Influence of dietary cholesterol on metabolic syndrome risk in middle-aged Korean adults: using the Korean Genome and Epidemiology Study (KoGES)

**DOI:** 10.1186/s12944-024-02271-1

**Published:** 2024-09-27

**Authors:** Hyunkyung Kwon, Minji Kang, Hyunjung Lim

**Affiliations:** 1 Department of Medical Nutrition, Graduate School of East-West Medical Science, Kyung Hee University, Yongin, South Korea; 2 Research Institute of Medical Nutrition, Kyung Hee University, Seoul, South Korea

**Keywords:** Dietary cholesterol, Metabolic syndrome, KoGES, HEXA study

## Abstract

**Background:**

The association between dietary cholesterol and metabolic diseases remains controversial. However, the majority of studies focus on egg intake, and there is a limitation in the availability of prospective cohort studies. Our study examined the association between dietary cholesterol and the incidence risk of metabolic syndrome (MetS) in middle aged adults using large prospective cohort study in Republic of Korea.

**Methods:**

The Health Examinees cohort from the Korean Genome and Epidemiology Study was used from baseline to follow-up. Dietary cholesterol intake was assessed by the validated semi-quantitative food frequency questionnaire. Participants were classified as quintile groups according to adjusted dietary cholesterol for total energy intake. MetS was defined as more than 3 of the 5 components of MetS. Hazard ratio (HR) and 95% confidence intervals (CI) for MetS were evaluated by multivariable cox regression analyses.

**Results:**

Of the total 40,578 participants, metabolic syndrome developed in 4,172 (10.28%) individuals during an average follow-up period of approximately 4.76 years. Dietary cholesterol did not exhibit a significant association with the risk of MetS after adjusting for potential confounding factors, but a trend was observed indicating an increased risk with higher intake (*p* for trend = 0.044). Among the components of MetS, the incidence risk of high waist circumference (HR: 1.164, 95% CI: 1.049–1.290), high blood pressure (HR: 1.188, 95% CI: 1.075–1.313), high serum triglyceride (HR: 1.132, 95% CI: 1.044–1.227) and high fasting blood glucose (HR: 1.217, 95% CI: 1.132–1.308) in the group that consumed the highest dietary cholesterol intake was increased compared with the group that consumed the lowest dietary cholesterol intake. Dose-response relationship suggested a positive linear association between dietary cholesterol intake and the risk of high waist circumference (*p*-linearity = 0.004), blood pressure (*p*-linearity = 0.012), and triglycerides (*p*-linearity = 0.005).

**Conclusion:**

This study suggests a positive association between dietary cholesterol intake and the risk of MetS and its components (abdominal obesity, hypertension, hypertriglyceridemia, and hyperglycemia) in middle-aged Korean adults.

**Supplementary Information:**

The online version contains supplementary material available at 10.1186/s12944-024-02271-1.

## Introduction

Metabolic syndrome (MetS) is a complex of risk factors for chronic diseases characterized by abdominal obesity, hyperglycemia, hypertension, and dyslipidemia [1]. The prevalence of MetS in the Republic of Korea has increased from 19.5% in 2014 to 22.9% in 2018, and the main components of MetS have been changing over the same period [2]. Specifically, the prevalence of low high-density lipoprotein cholesterolemia decreased, while the rates of abdominal obesity, high blood pressure (BP), and high blood glucose increased [2]. To prevent MetS, it is important to adjust modifiable lifestyle habits, especially diet, that can improve its components [3].

Many previous studies have shown that the excessive intake of dietary cholesterol (or egg) may contribute to the risk of type 2 diabetes mellitus (T2DM), dyslipidemia, hypertension, and cardiovascular disease [4–7]. The 2020 Dietary Reference Intakes for Koreans recommends that dietary cholesterol be consumed at less than 300 mg/day as it is determined that the effect of dietary cholesterol on the risk of chronic diseases cannot be overlooked [8].

However, the impact of dietary cholesterol on metabolic diseases remains inconsistent, with some studies reporting that the intake of dietary cholesterol (or eggs) did not significantly affect the development of chronic diseases [9, 10]. The 2015–2020 Dietary Guidelines for Americans eliminated the upper intake limit (less than 300 mg/day) due to the absence of a clear basis for the necessity to restrict dietary cholesterol [11].

Currently, the focus of most dietary cholesterol studies has been on eggs [12]. It has been reported that the lack of effect of dietary cholesterol on metabolic disease risk may be attributed to the antioxidants present in eggs [12, 13]. Furthermore, the majority of studies examining the association between dietary cholesterol and MetS have been conducted as cross-sectional studies, with limited availability of large prospective studies [14]. Therefore, we aim to investigate the relationship between dietary cholesterol and the risk of developing MetS and its five components in middle-aged adults using data from a large prospective cohort in Republic of Korea.

## Methods

### Study design and data source

This study relied on data from 2004 to 2016 from the Health Examinees study (HEXA study) of Korean Genome and Epidemiology Study (KoGES). The KoGES is a prospective cohort project conducted by the Korea National Institute of Health to investigate a scientific basis for risk factors of chronic diseases for Koreans [15]. The HEXA study, one of the various cohorts in KoGES, is a large-scale national cohort data that included over 40 years of participants recruited from 39 major and local health examination centers for health checkups [16]. The baseline survey was conducted from 2004 to 2013, and the follow-up survey from 2012 to 2016. Following standardized criteria, participants provided information about their socio-demographic characteristics, medical history, lifestyle factors, dietary intake, and physical activities. They also underwent physical examinations, which were consistently conducted by well-trained staff, and blood samples were collected for clinical blood tests. The detailed research protocols are outlined in previous studies [16].

All study participants gave written informed consent before entering the study. This study was approved by Kyung Hee University Institutional Review Board (KHGIRB-21-487).

### Study population

Among the 173,202 participants in the HEXA study who completed the baseline survey, 65,611 individuals who completed the follow-up survey were selected as the study population. From this group, individuals were excluded from the analysis if their data were missing or presented difficulties for analysis as follows: who were under 40 years or over 64 years (*n* = 7,088), who did not conduct a food intake frequency survey (*n* = 817), who consumed daily caloric intake is less than 500 kcal or more than 5000 kcal as a result of a dietary intake survey (*n* = 217), who did not have anthropometric, biochemical or socio-demographic data (*n* = 9,211) or who was classified as MetS in the baseline survey (*n* = 7,700). Ultimately, a total of 40,578 individuals (12,195 males and 28,383 females) with an average follow-up period of 4.76 years were included in the analysis.

### Dietary intake and classification of dietary cholesterol intake

The dietary intake of participants was investigated by a validated semi-quantitative food frequency questionnaire (SQ-FFQ) consisting of 106 food items [17]. The questionnaire on the frequency of consumption was classified according to nine options: never or seldom, once a month, 2–3 times/week, 1–2 times/week, 3–4 times/week, 5–6 times/week, once a day, 2 times/day, or > 3 times/day. The SQ-FFQ of the serving size part was classified according to small, medium, or large. The survey was conducted with trained interviewers, and formalized food photographs were used for accurate surveys.

The daily nutrient intake was estimated using a food composition Table [18]. The intake of calories and a total of 21 nutrients was assessed: carbohydrates, fiber, protein, fat, cholesterol, vitamin A, retinol, carotene, vitamin B_1_, vitamin B_2_, vitamin B_6_, folate, niacin, vitamin C, vitamin E, calcium, phosphorus, iron, sodium, potassium, and zinc. The calculated total dietary cholesterol intake was converted to intake per 1,000 kcal to adjust for total energy intake [19]. The adjusted total dietary cholesterol intake was categorized into quintiles (Q) based on the intake levels: Q1 (0.00–52.41 mg/1,000 kcal), Q2 (52.41–74.27 mg/1,000 kcal), Q3 (74.27–97.41 mg/1,000 kcal), Q4 (97.41–131.10 mg/1,000 kcal), Q5 (130.10–802.58 mg/1,000 kcal).

### Diagnosis of MetS

Metabolic syndrome was diagnosed through physical examinations and clinical blood tests conducted at the follow-up survey. Physical examinations such as waist circumference (WC) and BP were measured by experienced medical staff. Blood samples were collected, with at least 19 cc drawn into one serum separator tube (SST) and two ethylene-diamine-tetra-acetic acid (EDTA) tubes, then refrigerated. The samples were delivered to the laboratory within 24 h and analyzed using various laboratory tests to assess liver function (aspartate aminotransferase, alanine transaminase, albumin), lipid profile (total cholesterol, serum triglyceride (TG), serum high-density lipoprotein cholesterol (HDL-C), serum low-density lipoprotein cholesterol (LDL-C), and other parameters (blood urea nitrogen, creatinine, glucose).

According to the 2009 Joint Interim Statement [1], MetS was diagnosed if participants have more than 3 of the following 5 criteria; (1) WC ≥ 90 cm in males or ≥ 85 cm in females as modified by Korean Society for the Study of Obesity, (2) BP ≥ 130/85 mmHg or treatment for hypertension, (3) fasting blood glucose (FBG) ≥ 100 mg/dL or use of antihyperglycemic medication or treatment for T2DM; (4) TG ≥ 150 mg/dL or treatment for dyslipidemia; (5) serum high-density lipoprotein cholesterol (HDL-C) < 40 mg/dL in males or < 50 mg/dL in females. For WC, BP, FBG, and TG, higher levels than the respective diagnostic criteria were classified as high WC, high BP, high FBG, and high TG, respectively. HDL-C was classified as low HDL-C for levels lower than the diagnostic criteria.

### Assessment of other variables

Socio-demographic variables included education and household income level. The education level was classified into 3 categories: ‘High school graduate or less’, ‘College graduate’, ‘Graduate school or more’. The household income level classified into four categories according to self-reported average monthly income: ‘< 2,000,000 won/month’, ‘2,000,000 ∼ 4,000,000 won/month’, ‘4,000,000 ∼ 6,000,000 won/month’, ‘≥ 6,000,000 won/month’.

Lifestyle variables included smoking, alcohol and physical activity. Smoking status was classified as never smoking experience (‘never’), not currently smoking but having experience (‘past smoker’), and currently smoking (‘current smoker’). Drinking alcohol status was categorized as never drinking alcohol experience (‘never’), not currently drinking alcohol but having experience (‘past drinking alcohol’), and currently drinking alcohol (‘current drinking alcohol’). Physical activity was classified according to the frequency of exercise per week. When asked “Do you exercise regularly enough to make your body sweat?” “No” was classified as not exercising (‘0 times’) and “Yes” was classified into the following four categories according to the number of exercises per week: ‘1–2 times’, ‘3–4 times’, ‘5–6 times’ ‘Everyday’.

The height and weight of the participants were measured by experienced medical staff. Body mass index was determined by dividing the weight in kilograms by the height in meters squared.

### Statistical analysis

The general characteristics of participants were summarized as age, smoking status, drinking alcohol, physical activity, household income, educational level, anthropometrics, and biochemical data. Chi-squared test for categorical variables and ANOVA test and PROC GLM procedure for continuous variables were used. The hazard ratios (HR) at 95% confidence intervals (CI) were analyzed by conducting Cox regression to identify the association between dietary cholesterol and MetS or components of MetS. Model 1 was adjusted by age, sex, and daily energy intake. Model 2 was adjusted by age, sex, daily energy intake, smoking, drinking status, and exercise level. Model 3 was adjusted by age, sex, daily energy intake, smoking, drinking status, exercise level, income level and educational level. The linear trend between dietary cholesterol intake and the risk of metabolic syndrome was evaluated by calculating the *p*-value for the trend. In addition, dose-response relationships were examined using Cox proportional hazards models to identify both linear and potential nonlinear associations between dietary cholesterol intake and the risk of metabolic syndrome and its components. All statistical analyses were performed by SAS version 9.4 (SAS Institute, Cary, NC, USA) and R version 4.4.0 (RStudio, PBC, Boston, MA, USA). *P* values were considered significant if *p* < 0.05.

## Results

A total of 40,578 subjects with an average follow-up period of 4.76 years were included in the final analysis. The average age of all subjects was 51.58 years, with 30.05% (*n* = 12,195) for males and 69.95% (*n* = 28,383) for females. The average intake of dietary cholesterol by quintile was Q1: 36.30 mg/1,000 kcal (median 38.37 mg/1,000 kcal), Q2: 63.54 mg/1,000 kcal (median 63.69 mg/1,000 kcal), Q3: 85.33 mg/1,000 kcal (median 85.06 mg/1,000 kcal), Q4: 112.74 mg/1,000 kcal (median 112.13 mg/1,000 kcal) and Q5: 177.46 mg/1,000 kcal (median 163.51 mg/1,000 kcal) (Table 1). Overall, those with high dietary cholesterol intake had a high percentage of those with higher household income, education level, smokers, experience of drinking alcohol, and exercise frequency.

**Table 1:** General characteristics at baseline according to quintiles of dietary cholesterol among middle-aged Korean adults^1^

Variables		Dietary cholesterol intake					
		Q1	Q2	Q3	Q4	Q5	*P*-value^2^
Dietary cholesterol intake, mg/1,000 kcal							**< 0.001**
	Mean ± SD	36.30 ± 11.57	63.54 ± 6.26	85.33 ± 6.69	112.74 ± 9.48	177.46 ± 49.17	
	Median	38.37	63.69	85.06	112.13	163.51	
	Range	0.00–52.41	52.41–74.27	74.27–97.41	97.41–131.10	131.10–802.58	
Participants, n		8,115	8,116	8,116	8,116	8,115	
Age, years^3^		52.93 ± 6.64^a^	51.81 ± 6.76^b^	51.26 ± 6.79^c^	50.98 ± 6.87^c^	50.95 ± 6.94^c^	**< 0.001**
Males		2,581 (31.81)	2,604 (32.08)	2,573 (31.70)	2,362 (29.10)	2,075 (25.57)	**< 0.001**
Females		5,534 (68.19)	5,512 (67.92)	5,543 (68.30)	5,754 (70.90)	6,040 (74.43)	
Household income, won/month							
	< 2,000,000	2,750 (33.89)	2,204 (27.16)	1,926 (23.73)	1,801 (22.19)	1,767 (21.77)	**< 0.001**
	2,000,000 ∼ 4,000,000	3,489 (42.99)	3,646 (44.92)	3,776 (46.53)	3,880 (47.81)	3,800 (46.83)	
	4,000,000 ∼ 6,000,000	1,293 (15.93)	1,537 (18.94)	1,628 (20.06)	1,632 (20.11)	1,687 (20.79)	
	≥ 6,000,000	583 (7.18)	729 (8.98)	786 (9.68)	803 (9.89)	861 (10.61)	
Education level							
	High school graduate or less	6,199 (76.39)	5,760 (70.97)	5,456 (67.23)	5,232 (64.47)	5,066 (62.43)	
	College graduate	1,569 (19.33)	1,955 (24.09)	2,178 (26.84)	2,407 (29.66)	2,510 (30.93)	**< 0.001**
	Graduate school or more	347 (4.28)	401 (4.94)	482 (5.94)	477 (5.88)	539 (6.64)	
Smoking status							
	Never	6,355 (78.31)	6,248 (76.98)	6,265 (77.19)	6,240 (76.89)	6,101 (75.18)	**< 0.001**
	Past smoker	1,084 (13.36)	1,124 (13.85)	1,064 (13.11)	1,070 (13.18)	1,147 (14.13)	
	Current smoker	676 (8.33)	744 (9.17)	787 (9.70)	806 (9.93)	867 (10.68)	
Drinking alcohol status							
	Never	4,667 (57.51)	4,297 (52.94)	4,143 (51.05)	4,026 (49.61)	4,018 (49.51)	**< 0.001**
	Past drinking alcohol	293 (3.61)	258 (3.18)	237 (2.92)	225 (2.77)	260 (3.20)	
	Current drinking alcohol	3,155 (38.88)	3,561 (43.88)	3,736 (46.03)	3,865 (47.62)	3,837 (47.28)	
Physical activity, per week							
	0 times	3,831 (47.21)	3,564 (43.91)	3,549 (43.73)	3,503 (43.16)	3,387 (41.74)	**< 0.001**
	1–2 times	1,240 (15.28)	1,374 (16.93)	1,391 (17.14)	1,338 (16.49)	1,272 (15.67)	
	3–4 times	1,552 (19.13)	1,703 (20.98)	1,718 (21.17)	1,687 (20.79)	1,751 (21.58)	
	5–6 times	855 (10.54)	836 (10.30)	860 (10.60)	916 (11.29)	983 (12.11)	
	Everyday	637 (7.85)	639 (7.87)	598 (7.37)	672 (8.28)	722 (8.90)	

Q: quintile; SD: standard deviation

^1^ The values are presented as mean ± SD for continuous variables and n, percent (%) for categorical variables

^2^ Chi-squared test for categorical variables, and ANOVA test for continuous variables were used to compute the *p*-values (*p* < 0.05)

^3^ The values with different superscript letters represent the results of the post-hoc test (Scheffe; a > b > c)

Among quintile, only Q5 exceeded 300 mg of recommended intake of dietary cholesterol proposed by the 2020 Dietary Reference Intakes for Koreans [8] (Table 2). Total energy intake tended to increase as dietary cholesterol intake increased (*p* for trend < 0.001). As dietary cholesterol intake increased, the ratio of fat and protein intake increased and the ratio of carbohydrates decreased. Intake of other nutrients, including sodium, also tended to increase with increasing dietary cholesterol intake (all, *p* for trend < 0.001).

**Table 2:** Daily nutrients intake at baseline according to quintiles of dietary cholesterol among middle-aged Korean adults

Variables		Dietary cholesterol intake					
		Q1	Q2	Q3	Q4	Q5	*P*for trend^2^
Energy, kcal/day		1,576.53 ± 427.25^1^	1,706.23 ± 448.54	1,774.60 ± 489.65	1,845.51 ± 540.24	1,864.21 ± 637.08	**< 0.001**
	C: P:F ratio	78.5 : 11.0 : 8.9	74.6 : 12.3 : 11.9	71.6 : 13.3 : 14.1	68.7 : 14.4 : 16.2	64.7 : 16.1 : 18.8	
Carbohydrate, g/day		337.78 ± 0.28	325.43 ± 0.28	313.50 ± 0.28	300.72 ± 0.28	281.07 ± 0.28	**< 0.001**
Fiber, g/day		5.46 ± 0.03	5.64 ± 0.02	5.76 ± 0.02	5.87 ± 0.02	6.20 ± 0.02	**< 0.001**
Protein, g/day		49.91 ± 0.10	54.09 ± 0.10	58.46 ± 0.10	63.19 ± 0.10	71.48 ± 0.10	**< 0.001**
Fat, g/day		19.79 ± 0.10	23.95 ± 0.10	27.78 ± 0.10	31.74 ± 0.10	37.29 ± 0.10	**< 0.001**
Cholesterol, mg/day		77.17 ± 0.55	113.67 ± 0.54	149.31 ± 0.54	198.08 ± 0.54	314.85 ± 0.55	**< 0.001**
Vitamin A, mgRE/day		376.50 ± 3.04	424.80 ± 3.01	470.32 ± 3.01	514.08 ± 3.02	617.68 ± 3.02	**< 0.001**
Retinol, μg/day		36.56 ± 0.47	51.88 ± 0.46	64.98 ± 0.46	81.65 ± 0.46	121.69 ± 0.46	**< 0.001**
Carotene, μg/day		1,993.04 ± 17.78	2,181.29 ± 17.59	2,364.87 ± 17.58	2,515.36 ± 17.63	2,880.47 ± 17.66	**< 0.001**
Vitamin B_1_, mg/day		0.89 ± 0.00	0.95 ± 0.00	1.00 ± 0.00	1.05 ± 0.00	1.12 ± 0.00	**< 0.001**
Vitamin B_2_, mg/day		0.72 ± 0.00	0.82 ± 0.00	0.90 ± 0.00	0.98 ± 0.00	1.13 ± 0.00	**< 0.001**
Vitamin B_6_, mg/day		1.41 ± 0.00	1.50 ± 0.00	1.57 ± 0.00	1.65 ± 0.00	1.80 ± 0.00	**< 0.001**
Folate, μg/day		195.31 ± 1.01	207.30 ± 1.00	217.87 ± 1.00	227.18 ± 1.00	249.51 ± 1.00	**< 0.001**
Niacin, mg/day		12.59 ± 0.03	13.49 ± 0.03	14.47 ± 0.03	15.41 ± 0.03	16.83 ± 0.03	**< 0.001**
Vitamin C, mg/day		96.77 ± 0.65	103.88 ± 0.64	109.64 ± 0.64	113.21 ± 0.64	122.02 ± 0.64	**< 0.001**
Vitamin E, mg/day		6.96 ± 0.03	7.59 ± 0.03	8.16 ± 0.03	8.67 ± 0.03	9.66 ± 0.03	**< 0.001**
Calcium, mg/day		345.74 ± 2.08	402.08 ± 2.05	448.66 ± 2.05	490.02 ± 2.06	573.24 ± 2.06	**< 0.001**
Phosphorus, mg/day		765.17 ± 1.66	829.08 ± 1.65	886.49 ± 1.64	945.89 ± 1.65	1,056.85 ± 1.65	**< 0.001**
Iron, mg/day		8.68 ± 0.03	9.30 ± 0.03	9.92 ± 0.03	10.56 ± 0.03	11.96 ± 0.03	**< 0.001**
Sodium, mg/day		2,157.91 ± 12.70	2,320.78 ± 12.56	2,438.36 ± 12.56	2,545.10 ± 12.60	2,796.72 ± 12.61	**< 0.001**
Potassium, mg/day		1,933.53 ± 7.66	2,119.85 ± 7.58	2,264.13 ± 7.58	2,391.09 ± 7.60	2,628.83 ± 7.61	**< 0.001**
Zinc, μg/day		7.15 ± 0.02	7.50 ± 0.02	7.89 ± 0.02	8.32 ± 0.02	9.10 ± 0.02	**< 0.001**

Q: quintile; C:P: F: ratio of carbohydrate to protein to fat

^1^ The values are presented as lsmean ± standard error adjusted for total energy intake using multivariable general linear model

^2^ Bold-faced *p*-values indicate statistical significance

Table 3 shows the anthropometric and biochemical data and the proportion of MetS risk factors according to dietary cholesterol. WC, systolic blood pressure (SBP), TG, HDL-C and FBG were significantly increased during the follow-up period in all groups (all, *p* < 0.005). Among the components of MetS, the prevalence of high WC, high TG, and high FBG was elevated in all groups (*p* < 0.005). The highest increase in prevalence among components of MetS was high FBG.

**Table 3:** Change of MetS factors at baseline and follow-up study according to quintiles of dietary cholesterol among middle-aged Korean adults

Variables	Dietary cholesterol intake																								
	Q1						Q2					Q3			Q4							Q5			
	baseline			follow-up		baseline			follow-up		baseline			follow-up			baseline		follow-up		baseline			follow-up	
Anthropometrics																									
BMI, kg/m^2^	23.47 ± 0.03^1,2^		23.49 ± 0.03		23.51 ± 0.03			23.56 ± 0.03^**^		23.53 ± 0.03			23.61 ± 0.03^**^			23.46 ± 0.03		23.55 ± 0.03^**^		23.50 ± 0.03			23.56 ± 0.03^**^		
WC, cm	80.08 ± 0.08		80.54 ± 0.08^**^		80.21 ± 0.08			80.78 ± 0.08^**^		80.28 ± 0.08			80.93 ± 0.08^**^			80.08 ± 0.08		80.81 ± 0.08^**^		80.14 ± 0.08			80.66 ± 0.08^**^		
SBP, mmHg	121.24 ± 0.15		122.10 ± 0.16^**^		120.59 ± 0.15			121.86 ± 0.16^**^		120.64 ± 0.15			121.90 ± 0.16^**^			120.31 ± 0.15		121.65 ± 0.16^**^		119.84 ± 0.15			121.57 ± 0.16^**^		
DBP, mmHg	75.56 ± 0.10		74.82 ± 0.11^**^		75.21 ± 0.10			74.79 ± 0.11^**^		75.21 ± 0.10			74.72 ± 0.11^**^			74.99 ± 0.10		74.64 ± 0.11^*^		74.95 ± 0.10			74.46 ± 0.11^**^		
Biochemistry																									
Albumin, g/dL	4.64 ± 0.00		4.64 ± 0.00^**^		4.64 ± 0.00			4.64 ± 0.00^*^		4.64 ± 0.00			4.64 ± 0.00^**^			4.63 ± 0.00		4.65 ± 0.00^**^		4.64 ± 0.00			4.64 ± 0.00^**^		
AST, U/L	23.40 ± 0.13		25.03 ± 0.13^**^		23.25 ± 0.13			24.76 ± 0.13^**^		23.05 ± 0.13			24.76 ± 0.13^**^			23.16 ± 0.13		24.76 ± 0.13^**^		23.14 ± 0.13			24.79 ± 0.13^**^		
ALT, U/L	21.91 ± 0.18		22.53 ± 0.17^**^		21.99 ± 0.18			22.31 ± 0.17^*^		21.81 ± 0.18			22.66 ± 0.17^**^			21.89 ± 0.18		22.43 ± 0.17^**^		22.03 ± 0.18			22.54 ± 0.17^**^		
BUN, mg/dL	14.17 ± 0.04		14.85 ± 0.04^**^		14.34 ± 0.04			14.89 ± 0.04^**^		14.45 ± 0.04			14.94 ± 0.04^**^			14.61 ± 0.04		15.06 ± 0.04^**^		14.69 ± 0.04			15.03 ± 0.04^**^		
Cr, mg/dL	0.84 ± 0.00		0.82 ± 0.00^**^		0.84 ± 0.00			0.82 ± 0.00^**^		0.84 ± 0.00			0.82 ± 0.00^**^			0.84 ± 0.00		0.82 ± 0.00^**^		0.83 ± 0.00			0.82 ± 0.00^**^		
TG, mg/dL	111.13 ± 0.60		116.15 ± 0.61^**^		110.09 ± 0.60			115.53 ± 0.61^**^		109.04 ± 0.60			115.67 ± 0.61^**^			108.22 ± 0.60		114.42 ± 0.61^**^		107.90 ± 0.60			114.00 ± 0.61^**^		
TC, mg/dL	193.06 ± 0.39		198.17 ± 0.41^**^		195.29 ± 0.38			199.64 ± 0.40^**^		195.09 ± 0.38			200.57 ± 0.40^**^			196.12 ± 0.38		201.10 ± 0.40^**^		196.98 ± 0.38			201.36 ± 0.40^**^		
LDL-C, mg/dL	117.35 ± 0.35		116.95 ± 0.37^**^		119.00 ± 0.34			117.80 ± 0.37^*^		118.81 ± 0.34			118.62 ± 0.37			119.50 ± 0.34		118.82 ± 0.37		119.66 ± 0.34			118.51 ± 0.37		
HDL-C, mg/dL	53.49 ± 0.14		57.99 ± 0.17^**^		54.27 ± 0.14			58.73 ± 0.17^**^		54.46 ± 0.14			58.81 ± 0.17^**^			54.98 ± 0.14		59.39 ± 0.17^**^		55.74 ± 0.14			60.05 ± 0.17^**^		
FBG, mg/dL	92.15 ± 0.17		98.09 ± 0.18^**^		92.36 ± 0.17			97.99 ± 0.18^**^		92.49 ± 0.17			98.28 ± 0.18^**^			92.06 ± 0.17		98.01 ± 0.18^**^		92.41 ± 0.17			98.39 ± 0.18^**^		
MetS factors, n (%)																									
High WC^3^	1,098 (13.53)		1,401 (17.26) ^**^		1,096 (13.50)			1,457 (17.95) ^**^		1,087 (13.39)			1,502 (18.51) ^**^			1,076 (13.26)		1,458 (17.96) ^**^		1,126 (13.88)			1,446 (17.82) ^**^		
High BP^4^	1,555 (19.16)		1,367(16.85) ^**^		1,360 (16.76)			1,304 (16.07)		1,363 (16.79)			1,290 (15.89)			1,268 (15.62)		1,270 (15.65)		1,243 (15.32)			1,242 (15.30)		
High TG^5^	1,479 (18.23)		2,005 (24.71) ^**^		1,397 (17.21)			1,905 (23.47) ^**^		1,343 (16.55)			1,882 (23.19) ^**^			1,321 (16.28)		1,892 (23.31) ^**^		1,312 (16.17)			1,873 (23.08) ^**^		
Low HDL-C^6^	1,907 (23.50)		1,503 (18.52) ^**^		1,728 (21.29)			1,313 (16.18) ^**^		1,732 (21.34)			1,344 (16.56) ^**^			1,634 (20.13)		1,281 (15.78) ^**^		1,492 (18.39)			1,186 (14.61) ^**^		
High FBG^7^	1,227 (15.12)		2,448 (30.17) ^**^		1,212 (14.93)			2,441 (30.08) ^**^		1,193 (14.70)			2,401 (29.58) ^**^			1,156 (14.24)		2,385 (29.39) ^**^		1,188 (14.64)			2,474 (30.49) ^**^		

MetS: metabolic syndrome; Q: quintile; BMI: body mass index; WC: waist circumference; SBP: systolic blood pressure; DBP: diastolic blood pressure; ALT: alanine aminotransferase; AST: aspartate aminotransferase; BUN: blood urea nitrogen; Cr: serum creatinine; TG: serum triglyceride; TC: serum total cholesterol; LDL-C: serum low density lipoprotein-cholesterol; HDL-C: serum high density lipoprotein-cholesterol; FBG: fasting blood glucose

^1^ The values are presented as lsmean ± standard error adjusted for age and sex using general linear model

^2^ Paired t-test for continuous variables and chi-squared test for categorical variables were used to compute the *p* values (*p < 0.05, **p < 0.005)

^3^ High WC indicates more than 90 cm in males and more than 85 cm in females

^4^ High BP indicates more than 130/85 mmHg or treatment for hypertension

^5^ High TG indicates more than 150 mg/dL or treatment for dyslipidemia

^6^ Low HDL-C indicates less than 40 mg/dL in males and less than 50 mg/dL in females

^7^ High FBG indicates more than 100 mg/dL or use of antihyperglycemic medication or treatment for type 2 diabetes

The incidence of MetS according to dietary cholesterol intake is presented in Table 4. A total of 4,172 (10.28%) patients with MetS occurred during 193,188.1 person-years of follow-up study. After adjusting for age, sex, and daily energy intake (Model 1), the risk of MetS in the Q5 significantly increased by 10.8% (HR: 1.108, 95% CI: 1.005–1.222) compared with Q1 (*p* for trend = 0.036). After adjusting socio-demographic and lifestyle variables, the risk of MetS in Q5 was no longer statistically significant (HR: 1.103, 95% CI: 0.999–1.217) but showed that an increasing tendency remained significantly according to dietary cholesterol intake (*p* for trend = 0.044). In the subgroup analysis by sex, only men showed a tendency for an increased risk of MetS with higher quintiles of dietary cholesterol intake (*p* for trend = 0.024; Supplementary Table 1).

**Table 4:** Hazard ratio and 95% CI for risk of MetS according to quintiles of dietary cholesterol among middle-aged Korean adults

Variables	Dietary cholesterol intake					
	Q1	Q2	Q3	Q4	Q5	*P*for trend^4^
Total, n	8,115	8,116	8,116	8,116	8,115	
Cases, n (%)	875 (10.78)	836 (10.30)	836 (10.30)	820 (10.10)	805 (9.92)	
Person-years of follow up	39,759.2	38,838.6	38,543.8	38,329.1	37,717.4	
Event rates per 10,000	220.1	215.2	216.9	213.9	213.4	
HR (95% CI)						
Crude	Ref.	1.031 (0.937–1.133)	1.069 (0.972–1.175)	1.076 (0.978–1.184)	1.094 (0.994–1.205)	
Model 1^1^	Ref.	1.046 (0.951–1.151)	1.087 (0.988–1.196)	1.095 (0.994–1.207)	**1.108** **(1.005–1.222)**	**0.036**
Model 2^2^	Ref.	1.038 (0.943–1.142)	1.078 (0.980–1.187)	1.083 (0.983–1.194)	1.089 (0.987–1.201)	0.079
Model 3^3^	Ref.	1.042 (0.947–1.147)	1.087 (0.987–1.196)	1.095 (0.993–1.207)	1.103 (0.999–1.217)	**0.044**

MetS: metabolic syndrome; HR: hazard ratio; CI: confidence intervals; Q: quintile

^1^ Model 1 was adjusted age, sex and daily energy intake

^2^ Model 2 was adjusted age, sex, daily energy intake, smoking status, drinking alcohol status and exercise level

^3^ Model 3 was adjusted age, sex, daily energy intake, smoking status, drinking alcohol status, exercise level, income level and educational level

^4^ Bold-faced *p*-values indicate statistical significance

**Fig. 1:**
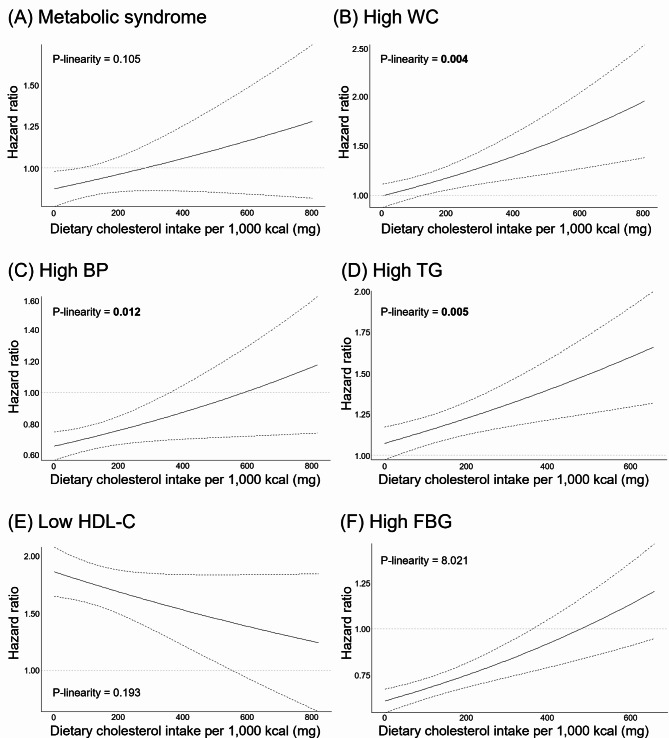
Dose-response relationships of dietary cholesterol intake with metabolic syndrome components. (**A**) ~ (**F**) represents the dose-response curve for the risk of metabolic syndrome and metabolic syndrome components according to dietary cholesterol intake. Hazard ratio (solid line) and 95% CI (dashed line) were adjusted for age, sex, daily energy intake, smoking status, drinking alcohol status, exercise level, income level and educational level. The average dietary cholesterol intake of 36.3 mg/1000 kcal in the lowest quintile group was set as reference WC: waist circumference; BP: blood pressure; TG: serum triglyceride; HDL-C: serum high density lipoprotein-cholesterol; FBG: fasting blood glucose; CI: confidence intervals

In terms of components of metabolic syndrome, high intake of dietary cholesterol has been shown to be positively associated with the risk of most components except for low HDL-C (Table 5). After adjusting for all covariates (Model 3), Q5 with the highest dietary cholesterol intake had a higher risk of high WC (HR: 1.164, 95% CI: 1.049–1.290), high BP (HR: 1.188, 95% CI: 1.075–1.313), high TG (HR: 1.132, 95% CI: 1.044–1.227), and FBG (HR: 1.217, 95% CI: 1.132–1.308) than Q1 with the lowest intake before adjustment. As dietary cholesterol intake increased, the risk tended to increase (all, *p* for trend < 0.05). There was no association between dietary cholesterol intake and incidence risk of low HDL-C (*p* for trend = 0.605). Dose-response curves visually indicate that as dietary cholesterol intake increases, the risk of MetS and its components, except for HDL cholesterol, also increases (Fig. 1). Specifically, there is a positive linear association suggested for high WC (*p*-linearity = 0.004), high BP (*p*-linearity = 0.012), and high TG (*p*-linearity = 0.005).

**Table 5:** Hazard ratio and 95% CI for risk of MetS factors according to quintiles of dietary cholesterol among middle-aged Korean adults

Variables	HR (95% CI)					
	Q1	Q2	Q3	Q4	Q5	*p*for trend^4^
**High WC (*n* = 35**,**095)**^5,6^						
Crude	Ref.	**1.105 (1.000-1.221)**	**1.131 (1.024–1.250)**	1.095 (0.990–1.211)	**1.136 (1.027–1.256)**	
Model 1^1^		**1.125 (1.017–1.244)**	**1.156 (1.045–1.278)**	**1.121 (1.012–1.242)**	**1.160 (1.046–1.285)**	**0.022**
Model 2^2^		**1.120 (1.013–1.238)**	**1.152 (1.042–1.274)**	**1.115 (1.006–1.236)**	**1.153 (1.040–1.278)**	**0.030**
Model 3^3^		**1.122 (1.014–1.240)**	**1.156 (1.045–1.279)**	**1.124 (1.014–1.246)**	**1.164 (1.049–1.290)**	**0.018**
**High BP (*n* = 33**,**789)**^7^						
Crude	Ref.	1.094 (0.994–1.204)	1.082 (0.982–1.193)	**1.115 (1.012–1.228)**	**1.121 (1.017–1.236)**	
Model 1		**1.136 (1.031–1.251)**	**1.138 (1.031–1.256)**	**1.187 (1.076–1.310)**	**1.190 (1.077–1.314)**	**0.001**
Model 2		**1.131 (1.027–1.246)**	**1.130 (1.024–1.247)**	**1.174 (1.063–1.296)**	**1.175 (1.063–1.298)**	**0.004**
Model 3		**1.136 (1.031–1.251)**	**1.139 (1.032–1.257)**	**1.184 (1.072–1.307)**	**1.188 (1.075–1.313)**	**0.002**
**High TG (*n* = 33**,**726)**^8^						
Crude	Ref.	0.996 (0.920–1.077)	1.027 (0.950–1.111)	1.041 (0.962–1.127)	**1.091 (1.008–1.180)**	
Model 1		1.029 (0.950–1.113)	1.071 (0.989–1.159)	**1.099 (1.014–1.191)**	**1.144 (1.056–1.239)**	**< 0.001**
Model 2		1.021 (0.943–1.105)	1.065 (0.984–1.153)	**1.089 (1.004–1.180)**	**1.126 (1.039–1.221)**	**0.001**
Model 3		1.021 (0.943–1.105)	1.066 (0.984–1.155)	**1.092 (1.008–1.184)**	**1.132 (1.044–1.227)**	**0.001**
**Low HDL-C (*n* = 32**,**085)**^9^						
Crude	Ref.	0.890 (0.789–1.004)	0.962 (0.854–1.084)	0.906 (0.802–1.023)	0.920 (0.815–1.039)	
Model 1		0.902 (0.799–1.019)	0.980 (0.869–1.105)	0.929 (0.821–1.052)	0.942 (0.832–1.066)	0.543
Model 2		0.901 (0.798–1.018)	0.980 (0.869–1.105)	0.929 (0.821–1.052)	0.937 (0.827–1.060)	0.490
Model 3		0.903 (0.800–1.020)	0.985 (0.873–1.111)	0.937 (0.828–1.062)	0.945 (0.835–1.071)	0.605
**High FBG (***n*** = 34**,**602)**^10^						
Crude	Ref.	**1.081 (1.007–1.160)**	**1.098 (1.023–1.178)**	**1.121 (1.044–1.203)**	**1.185 (1.104–1.271)**	
Model 1		**1.110 (1.034–1.191)**	**1.129 (1.052–1.213)**	**1.171 (1.089–1.258)**	**1.239 (1.153–1.331)**	**< 0.001**
Model 2		**1.104 (1.029–1.185)**	**1.120 (1.043–1.203)**	**1.155 (1.074–1.241)**	**1.215 (1.131–1.306)**	**< 0.001**
Model 3		**1.105 (1.029–1.186)**	**1.121 (1.044–1.204)**	**1.156 (1.075–1.242)**	**1.217 (1.132–1.308)**	**< 0.001**

MetS: metabolic syndrome; HR: hazard ratio; CI: confidence intervals; Q: quintile; WC: waist circumference; BP: blood pressure; TG: serum triglyceride; HDL-C: serum high density lipoprotein-cholesterol; FBG: fasting blood glucose

^1^ Model 1 was adjusted age, sex and daily energy intake

^2^ Model 2 was adjusted age, sex, daily energy intake, smoking status, drinking alcohol status and exercise level

^3^ Model 3 was adjusted age, sex, daily energy intake, smoking status, drinking alcohol status, exercise level, income level and educational level

^4^ Bold-faced *p*-values indicate statistical significance

^5^ The numbers of participants represent the count of participants excluding those who already had the events at baseline for each variable. All groups share the same range for quintiles of dietary cholesterol intake: Q1 (0.00–52.41 mg/1,000 kcal), Q2 (52.41–74.27 mg/1,000 kcal), Q3 (74.27–97.41 mg/1,000 kcal), Q4 (97.41–131.10 mg/1,000 kcal), Q5 (130.10–802.58 mg/1,000 kcal)

^6^ High WC indicates more than 90 cm in males and more than 85 cm in females

^7^ High BP indicates more than 130/85 mmHg or treatment for hypertension

^8^ High TG indicates more than 150 mg/dL or treatment for dyslipidemia

^9^ Low HDL-C indicates less than 40 mg/dL in males and less than 50 mg/dL in females

^10^ High FBG indicates more than 100 mg/dL or use of antihyperglycemic medication or treatment for type 2 diabetes

Similarly, in the subgroup analysis by sex (Supplementary Tables 1–2), higher dietary cholesterol intake was associated with an increased risk of high WC (men, *p* for trend = 0.014; women, *p* for trend = 0.043), high TG (men, *p* for trend = 0.003; women, *p* for trend = 0.036), and high FBG (men, *p* for trend < 0.001; women, *p* for trend < 0.001). However, for high BP, a significant increasing trend was observed only in women (*p* for trend = 0.005; Supplementary Table 2).

## Discussion

In a large-scale prospective cohort study in the Republic of Korea, we identified a positive correlation between dietary cholesterol intake and an increased risk of high WC, high BP, high TG, and high FBG among the components of MetS. To the best of our knowledge, the present study is the first to investigate the association between dietary cholesterol intake and the incidence risk of MetS using prospective cohort data.

The average dietary cholesterol intake of all subjects in our study was 170.62 mg/day, which was lower than the cholesterol intake of adults aged 19 and older (267.4 mg/day) presented in the Korea Health Statistics 2021 [20]. This was also lower than the global average dietary cholesterol intake of 228 mg/day reported in the systematic analysis of 266 countries [21]. In 2019, the American Heart Association recommended a healthy diet pattern (Dietary Approaches to Stop Hypertension diet (DASH diet), Mediterranean diet) with significantly lower cholesterol [22]. This is slightly higher considering that the dietary cholesterol content of the DASH diet is about 150 mg/day (based on 2100 kcal) [3]. Within the quantiles of dietary cholesterol intake in our study, only the highest quintile (Q5) exceeded the recommended intake of dietary cholesterol (300 mg/day). Meanwhile, as dietary cholesterol intake increased, the intake of other nutrients (excluding carbohydrates), including calories, also tended to increase. Considering that cholesterol primarily comes from animal sources, the increase in energy intake may be attributed to the concurrent rise in fat consumption. As evidence supporting this, reports indicate a continuous increase in energy intake from animal foods, including meat, in Republic of Korea [23]. A 14-year follow-up study in China identified an elevated risk of hypertension and mortality in both the group with consistently high intake (> 300 mg/day) and the group with progressively increasing dietary cholesterol intake, despite starting with an intake below 200 mg/day [6]. Even if the current dietary cholesterol intake is low, there is potential for an increase, indicating the need for ongoing management.

Previous studies examining the association between dietary cholesterol (or eggs) and metabolic diseases have yielded inconsistent results. In contrast to the present study, in a cohort study of Finnish men, there was no change in BP according to dietary cholesterol intake [24]. In five years follow-up study for Japanese, dietary cholesterol was not associated with an increased risk of T2DM [25]. One of the reasons for this discrepancy is the different follow-up period for studies, which might be insufficient until chronic disease occurred. Also, there are other possible reasons that the incidence risk was significant in our study. MetS is a combination of symptoms prior to the onset of chronic disease [1], so a more relaxed criterion was used.

Some studies on dietary cholesterol (or eggs) and diseases related to MetS or its components have reported findings consistent with our results. This aligns with the findings of a cross-sectional study conducted in China, which examined the association between dietary cholesterol and metabolic syndrome [14]. The odds ratio of MetS in the highest dietary cholesterol group was 1.31 (CI: 1.12–1.53, *p* for trend = 0.005) compared to the lowest dietary cholesterol group [14]. Moreover, dietary cholesterol exhibited a significant impact on the elevation of SBP, blood glucose, WC, and serum HDL-C, all associated with MetS, mirroring our study findings [14]. In a multi-country cohort study, cholesterol intake was positively correlated with an elevated risk of hypertension after adjusting for covariates [26]. Additionally, a prospective cohort study in France revealed an association between eggs, dietary cholesterol intake, and an increased risk of hypertension [27].

In relation to blood glucose, a large cohort study of French women demonstrated a positive correlation between the incidence risk of T2DM and a high intake of dietary cholesterol [28]. Studies analyzed eggs and meat, which are the main sources of cholesterol, and confirmed that it was related to T2DM [29, 30]. High dietary cholesterol intake was linked to a decline in liver function, a significant site of cholesterol metabolism [31]. This suggests that dietary cholesterol intake may contribute to the risk of impaired liver function, potentially leading to insulin resistance, a major factor in the development of MetS [32].

There is possible evidence for our findings, although it was the result of animal experiments. In vivo, excessive dietary cholesterol intake increased the size of visceral fat [33]. Adipocyte hypertrophy can increase free cholesterol storage in adipose tissue, and consequently, it lowers glucose uptake and glut-4 expression, thereby reducing insulin sensitivity [34, 35]. This mechanism is related to insulin resistance and might even affect the development of chronic diseases such as hypertension and dyslipidemia [36]. Therefore, more research is needed in the future, but the effect of dietary cholesterol on the body cannot be completely overlooked.

The absence of an association with the risk of low HDL-C may be attributed to an increase in serum total cholesterol levels with the rise in dietary cholesterol intake. Several studies have reported that dietary cholesterol is associated with an increase in serum total cholesterol and serum LDL-C [37–40]. Furthermore, egg intake was linked to an increase in HDL-C and LDL-C without a significant alteration in the HDL-C: LDL-C ratio [41]. HDL-C may increase as the level of Apo A-1 in the blood rises, potentially attributed to a high-fat/high-cholesterol diet [41–43]. Hypercholesterolemia and hyper-LDL cholesterolemia are well known as major causes of cardiovascular diseases risk or atherosclerosis [44]. Hence, opting for dietary cholesterol intake is not recommended for enhancing HDL-C levels.

This study had significant strengths. First, a representative prospective cohort dataset was used. KoGES is a cohort established to identify the causes of chronic diseases in Koreans and recruited from various regions in Republic of Korea. Moreover, it is the initial study to analyze the relationship between dietary cholesterol and MetS among Koreans. Despite these strengths, there are some limitations. Firstly, a dietary survey was calculated based on the SQ-FFQ data, which might not be accurate to actual intake (or usual intake). Second, in this study, the average follow-up period was less than five years. Third, intake of saturated fatty acids often increases with dietary cholesterol, and such intake may be associated with MetS and its components. However, we could not control for the effects of saturated fatty acids because the relevant data were not collected in our dataset. Finally, potential issues include inverse causality and uncontrolled confounding factors that were not collected. To broaden the scope of research findings, future studies should analyze the long-term effects of dietary cholesterol intake on MetS.

## Conclusions

In conclusion, our findings indicate that individuals with high dietary cholesterol consumption face an elevated risk of abdominal obesity, hypertension, hypertriglyceridemia, and hyperglycemia. Therefore, excessive intake of dietary cholesterol increases the risk of each component of the MetS, which can lead to MetS, so prudent intake of dietary cholesterol is considered. It can be utilized as supporting evidence and serve as a reference when developing guidelines for dietary cholesterol consumption.

## Electronic supplementary material

Below is the link to the electronic supplementary material.

Supplementary Material 1

## Data Availability

The data used in this study are available after obtaining permission from National Institute of Health, Korea Disease Control and Prevention Agency, Republic of Korea.

## Abbreviations

BP: Blood pressure
CI: Confidence intervals
DASH diet: Dietary Approaches to Stop Hypertension diet
FBG: Fasting blood glucose
HDL-C: High-density lipoprotein cholesterol
HEXA study: Health Examinees study
HR: Hazard ratio
KoGES: Korean Genome and Epidemiology Study
MetS: Metabolic syndrome
Q: Quintiles
SBP: Systolic blood pressure
SQ-FFQ: Semi-quantitative food frequency questionnaire
T2DM: Type 2 diabetes mellitus
TG: Triglyceride
WC: Waist circumference
