# Supplementary material for: Influence of dietary cholesterol on metabolic syndrome risk in middle-aged Korean adults: using the Korean Genome and Epidemiology Study (KoGES)
Source: Lipids Health Dis. 2024 Sep 27;23:315. doi: 10.1186/s12944-024-02271-1 (PMC11438006; doi:10.1186/s12944-024-02271-1)
Supplement: Supplementary file 1 — Supplementary Material 1 [file 12944_2024_2271_MOESM1_ESM.docx]

Supplementary materials

**Supplementary Table 1.** Hazard ratio and 95% CI for risk of MetS and its factors according to quintiles of dietary cholesterol among middle-aged Korean men

| **Variables** | **HR (95% CI)** | | | | | | |
| --- | --- | --- | --- | --- | --- | --- | --- |
|  | **Q1** | | **Q2** | **Q3** | **Q4** | **Q5** | ***p* for trend**^4^ |
| Range of dietary cholesterol intake per 1000 kcal (mg/1000kcal) | 0.0 – 51.1 | | 51.1 – 71.8 | 71.8 – 92.7 | 92.7 – 123.8 | 123.9 – 802.6 |  |
| **Metabolic syndrome (n=12,195)**^5^ |  | |  |  |  |  |  |
| Crude | | Ref. | 1.031 (0.882-1.206) | 1.112 (0.952-1.298) | 1.130 (0.968-1.320) | **1.268 (1.089-1.476)** |  |
| Model 1^1^ | |  | 1.008 (0.861-1.180) | 1.078 (0.921-1.261) | 1.083 (0.924-1.269) | **1.211 (1.035-1.418)** | **0.008** |
| Model 2^2^ | |  | 0.991 (0.847-1.160) | 1.053 (0.899-1.232) | 1.057 (0.901-1.239) | **1.171 (1.001-1.371)** | **0.024** |
| Model 3^3^ | |  | 0.998 (0.853-1.168) | 1.053 (0.900-1.233) | 1.059 (0.903-1.242) | **1.175 (1.004-1.376)** | **0.024** |
| **High WC (n=10,136)**^6^ |  | |  |  |  |  |  |
| Crude | Ref. | | 1.149 (0.960-1.376) | **1.265 (1.057-1.514)** | **1.216 (1.013-1.460)** | **1.344 (1.123-1.608)** |  |
| Model 1 |  |  | 1.150 (0.960-1.377) | **1.265 (1.055-1.517)** | **1.211 (1.004-1.461)** | **1.335 (1.109-1.607)** | **0.004** |
| Model 2 |  |  | 1.135 (0.948-1.361) | **1.247 (1.040-1.496)** | 1.192 (0.988-1.438) | **1.311 (1.089-1.579)** | **0.008** |
| Model 3 |  |  | 1.134 (0.946-1.359) | **1.238 (1.032-1.485)** | 1.178 (0.976-1.422) | **1.292 (1.072-1.557)** | **0.014** |
| **High BP (n=9,435)**^7^ |  | |  |  |  |  |  |
| Crude | Ref. | | 1.027 (0.877-1.202) | 1.111 (0.949-1.301) | 1.142 (0.976-1.335) | 1.123 (0.960-1.315) |  |
| Model 1 |  |  | 1.039 (0.887-1.218) | 1.133 (0.966-1.328) | 1.169 (0.996-1.372) | 1.152 (0.980-1.355) | 0.054 |
| Model 2 |  |  | 1.033 (0.881-1.211) | 1.116 (0.951-1.310) | 1.145 (0.975-1.345) | 1.123 (0.955-1.322) | 0.115 |
| Model 3 |  |  | 1.042 (0.888-1.220) | 1.125 (0.959-1.321) | 1.152 (0.981-1.353) | 1.136 (0.965-1.337) | 0.092 |
| **High TG (n=9,105)**^8^ |  | |  |  |  |  |  |
| Crude | Ref. | | 1.072 (0.924-1.244) | **1.179 (1.019-1.365)** | **1.251 (1.081-1.447)** | **1.300 (1.124-1.505)** |  |
| Model 1 |  |  | 1.051 (0.905-1.220) | 1.146 (0.988-1.329) | **1.209 (1.041-1.403)** | **1.262 (1.085-1.466)** | **0.001** |
| Model 2 |  |  | 1.034 (0.890-1.201) | 1.118 (0.964-1.297) | **1.188 (1.023-1.379)** | **1.228 (1.057-1.428)** | **0.002** |
| Model 3 |  |  | 1.035 (0.891-1.203) | 1.112 (0.958-1.291) | **1.185 (1.020-1.376)** | **1.225 (1.053-1.424)** | **0.003** |
| **Low HDL-C (n=10,840)**^9^ |  | |  |  |  |  |  |
| Crude | Ref. | | 0.801 (0.624-1.029) | 0.928 (0.728-1.183) | 0.918 (0.718-1.172) | 1.042 (0.822-1.321) |  |
| Model 1 |  |  | 0.788 (0.612-1.013) | 0.904 (0.707-1.157) | 0.883 (0.687-1.135) | 0.997 (0.780-1.274) | 0.555 |
| Model 2 |  |  | 0.781 (0.606-1.004) | 0.899 (0.702-1.150) | 0.885 (0.688-1.138) | 1.000 (0.782-1.279) | 0.510 |
| Model 3 |  |  | 0.786 (0.611-1.012) | 0.906 (0.708-1.160) | 0.899 (0.699-1.156) | 1.011 (0.791-1.294) | 0.461 |
| **High FBG (n=9,407)**^10^ |  | |  |  |  |  |  |
| Crude | Ref. | | 1.115 (0.995-1.250) | 1.093 (0.975-1.226) | **1.223 (1.092-1.369)** | **1.250 (1.116-1.400)** |  |
| Model 1 |  |  | 1.119 (0.998-1.254) | 1.098 (0.978-1.233) | **1.229 (1.095-1.380)** | **1.255 (1.117-1.411)** | **< 0.001** |
| Model 2 |  |  | 1.110 (0.990-1.245) | 1.087 (0.968-1.221) | **1.209 (1.076-1.357)** | **1.227 (1.091-1.380)** | **< 0.001** |
| Model 3 |  |  | 1.112 (0.991-1.247) | 1.087 (0.967-1.221) | **1.207 (1.075-1.356)** | **1.227 (1.091-1.381)** | **< 0.001** |

MetS, metabolic syndrome; HR, hazard ratio; CI, confidence intervals; Q, quintile; WC, waist circumference; BP, blood pressure; TG, serum triglyceride; HDL-C, serum high density lipoprotein-cholesterol; FBG, fasting blood glucose.

^1^ Model 1 was adjusted age, sex and daily energy intake.

^2^ Model 2 was adjusted age, sex, daily energy intake, smoking status, drinking alcohol status and exercise level.

^3^ Model 3 was adjusted age, sex, daily energy intake, smoking status, drinking alcohol status, exercise level, income level and educational level.

^4^ Bold-faced *p*-values indicate statistical significance.

^5^ The numbers of participants represent the count of participants excluding those who already had the events at baseline for each variable. All groups share the same range for quintiles of dietary cholesterol intake.

^6^ High WC indicates more than 90 cm in males.

^7^ High BP indicates more than 130/85 mmHg or treatment for hypertension.

^8^ High TG indicates more than 150 mg/dL or treatment for dyslipidemia.

^9^ Low HDL-C indicates less than 40 mg/dL in males.

^10^ High FBG indicates more than 100 mg/dL or use of antihyperglycemic medication or treatment for type 2 diabetes.

**Supplementary Table 2.** Hazard ratio and 95% CI for risk of MetS and its factors according to quintiles of dietary cholesterol among middle-aged Korean women

| **Variables** | **HR (95% CI)** | | | | | | |
| --- | --- | --- | --- | --- | --- | --- | --- |
|  | **Q1** | | **Q2** | **Q3** | **Q4** | **Q5** | ***p* for trend**^4^ |
| Range of dietary cholesterol intake per 1000 kcal (mg/1000kcal) | 0.0 – 53.0 | | 53.0 – 75.4 | 75.4 – 99.5 | 99.5 – 134.2 | 134.2 – 655.0 |  |
| **Metabolic syndrome (n=28,383)**^5^ |  | |  |  |  |  |  |
| Crude | | Ref. | 1.033 (0.917-1.164) | 1.048 (0.929-1.181) | 1.046 (0.927-1.180) | 0.996 (0.880-1.128) |  |
| Model 1^1^ | |  | 1.082 (0.960-1.220) | 1.100 (0.974-1.241) | 1.114 (0.985-1.259) | 1.052 (0.928-1.193) | 0.483 |
| Model 2^2^ | |  | 1.082 (0.960-1.220) | 1.099 (0.974-1.241) | 1.107 (0.979-1.253) | 1.041 (0.917-1.181) | 0.614 |
| Model 3^3^ | |  | 1.087 (0.964-1.226) | 1.112 (0.985-1.256) | 1.125 (0.994-1.273) | 1.063 (0.936-1.207) | 0.394 |
| **High WC (n=24,959)**^6^ |  | |  |  |  |  |  |
| Crude | Ref. | | 1.099 (0.975-1.240) | 1.118 (0.992-1.260) | 1.071 (0.949-1.208) | 1.100 (0.973-1.242) |  |
| Model 1 |  |  | **1.137 (1.007-1.283)** | **1.157 (1.026-1.306)** | 1.119 (0.989-1.265) | **1.148 (1.014-1.299)** | 0.097 |
| Model 2 |  |  | **1.134 (1.005-1.281)** | **1.157 (1.025-1.306)** | 1.118 (0.988-1.264) | **1.143 (1.010-1.294)** | 0.112 |
| Model 3 |  |  | **1.140 (1.010-1.287)** | **1.170 (1.036-1.321)** | **1.139 (1.006-1.288)** | **1.172 (1.035-1.327)** | **0.043** |
| **High BP (n=24,354)**^7^ |  | |  |  |  |  |  |
| Crude | Ref. | | **1.132 (1.003-1.278)** | 1.069 (0.945-1.211) | 1.105 (0.977-1.249) | 1.121 (0.990-1.268) |  |
| Model 1 |  |  | **1.207 (1.069-1.363)** | **1.145 (1.011-1.298)** | **1.211 (1.069-1.373)** | **1.221 (1.076-1.384)** | **0.008** |
| Model 2 |  |  | **1.208 (1.069-1.364)** | **1.139 (1.005-1.291)** | **1.202 (1.061-1.363)** | **1.219 (1.075-1.383)** | **0.010** |
| Model 3 |  |  | **1.211 (1.071-1.368)** | **1.147 (1.012-1.301)** | **1.213 (1.070-1.376)** | **1.235 (1.088-1.401)** | **0.005** |
| **High TG (n=24,621)**^8^ |  | |  |  |  |  |  |
| Crude | Ref. | | 0.968 (0.882-1.062) | 0.973 (0.886-1.068) | 0.968 (0.881-1.063) | 1.018 (0.927-1.118) |  |
| Model 1 |  |  | 1.025 (0.934-1.125) | 1.043 (0.949-1.146) | 1.064 (0.967-1.171) | **1.105 (1.005-1.215)** | **0.028** |
| Model 2 |  |  | 1.024 (0.933-1.124) | 1.042 (0.948-1.145) | 1.059 (0.962-1.166) | 1.094 (0.994-1.203) | 0.052 |
| Model 3 |  |  | 1.025 (0.933-1.125) | 1.043 (0.949-1.147) | 1.064 (0.967-1.172) | 1.101 (1.000-1.212) | **0.036** |
| **Low HDL-C (n=21,245)**^9^ |  | |  |  |  |  |  |
| Crude | Ref. | | 0.913 (0.795-1.048) | 0.966 (0.842-1.107) | 0.894 (0.777-1.029) | 0.874 (0.759-1.008) |  |
| Model 1 |  |  | 0.943 (0.821-1.083) | 1.004 (0.875-1.152) | 0.943 (0.818-1.088) | 0.918 (0.795-1.061) | 0.276 |
| Model 2 |  |  | 0.943 (0.820-1.083) | 1.005 (0.876-1.154) | 0.940 (0.815-1.085) | 0.912 (0.789-1.054) | 0.233 |
| Model 3 |  |  | 0.944 (0.821-1.084) | 1.008 (0.878-1.158) | 0.946 (0.819-1.091) | 0.917 (0.793-1.060) | 0.273 |
| **High FBG (n=25,195)**^10^ |  | |  |  |  |  |  |
| Crude | Ref. | | 1.055 (0.964-1.153) | 1.088 (0.994-1.190) | 1.053 (0.961-1.153) | **1.142 (1.043-1.249)** |  |
| Model 1 |  |  | **1.113 (1.017-1.218)** | **1.154 (1.054-1.263)** | **1.138 (1.038-1.248)** | **1.229 (1.122-1.346)** | **< 0.001** |
| Model 2 |  |  | **1.109 (1.014-1.214)** | **1.146 (1.047-1.254)** | **1.123 (1.024-1.232)** | **1.208 (1.102-1.323)** | **< 0.001** |
| Model 3 |  |  | **1.109 (1.013-1.214)** | **1.145 (1.046-1.254)** | **1.124 (1.024-1.234)** | **1.210 (1.104-1.326)** | **< 0.001** |

MetS, metabolic syndrome; HR, hazard ratio; CI, confidence intervals; Q, quintile; WC, waist circumference; BP, blood pressure; TG, serum triglyceride; HDL-C, serum high density lipoprotein-cholesterol; FBG, fasting blood glucose.

^1^ Model 1 was adjusted age, sex and daily energy intake.

^2^ Model 2 was adjusted age, sex, daily energy intake, smoking status, drinking alcohol status and exercise level.

^3^ Model 3 was adjusted age, sex, daily energy intake, smoking status, drinking alcohol status, exercise level, income level and educational level.

^4^ Bold-faced *p*-values indicate statistical significance.

^5^ The numbers of participants represent the count of participants excluding those who already had the events at baseline for each variable. All groups share the same range for quintiles of dietary cholesterol intake.

^6^ High WC indicates more than 85 cm in females.

^7^ High BP indicates more than 130/85 mmHg or treatment for hypertension.

^8^ High TG indicates more than 150 mg/dL or treatment for dyslipidemia.

^9^ Low HDL-C indicates less than 50 mg/dL in females.

^10^ High FBG indicates more than 100 mg/dL or use of antihyperglycemic medication or treatment for type 2 diabetes.
